# Graphene nanomaterials: A new frontier in preventing respiratory fungal infections

**DOI:** 10.22034/cmm.2025.345248.1618

**Published:** 2025-05-23

**Authors:** Bahram Naderi-Nabi, Iman Haghani, Abbas Raeisabadi, Akbar Hoseinnejad, Hakimeh Sarshad, Nazanin Rahmanian, Mohammad Haghighi

**Affiliations:** 1 Anesthesiology Research Center, Department of Anesthesiology, Alzahra Hospital, Guilan University of Medical Sciences, Rasht, Iran; 2 Invasive Fungi Research Center, Communicable Diseases Institute, Mazandaran University of Medical Sciences, Sari, Iran; 3 Department of Medical Mycology, School of Medicine, Mazandaran University of Medical Sciences, Sari, Iran; 4 Student Research Committee, Ahvaz Jundishapur University of Medical Sciences, Ahvaz, Iran

**Keywords:** Antifungal agents, Nano-graphene oxide, Pulmonary fungal infections

## Abstract

Nano-graphene oxide is a promising *Candidate* for therapeutic applications, particularly due to its notable antifungal and antibacterial properties, which stem from the unique physicochemical characteristics of this innovative nanocarrier. Antifungal efficacy of nano-graphene oxide is increasingly attracting attention, particularly in light of the rising resistance of pathogens to conventional drug therapies. Upon exposure to graphene oxide, fungal cells generate reactive oxygen species, a key indicator of cellular oxidative stress. This mechanism accounts for the apoptotic-like cell death observed in the presence of graphene oxide. This nano-drug delivery system holds the potential to achieve therapeutic efficacy at reduced doses, minimize side effects, enable controlled drug release, prolong circulation time, reduce toxicity, and enhance the stability of the nano-formulation, particularly when administered as an inhaled dry powder. However, the factors influencing the antifungal activity of nano-graphene oxide and the underlying mechanisms remain poorly understood. This article aimed to review the anti-pathogenic properties of nano-graphene oxide, focusing on its antifungal mechanisms and its role in biofilm formation associated with pulmonary fungal infections.

## Introduction

Fungal respiratory infections represent an escalating concern and a major cause of mortality in immunocompromised patients [ 1 ,  2 ]. The most common fungal pneumonia etiological agents include *Aspergillus*, *Mucorales*, *Cryptococcus*, and endemic dimorphic fungi. [ 3 ,  4 ]. The genus *Aspergillus* from the division *Ascomycota* is among the most prevalent and opportunistic molds worldwide [ 5 ]. The attributable mortality rate of pneumonia caused by *Aspergillus* is more than 80% [ 6 ]. Several species of *Aspergillus* spp., notably *A. fumigatus*, *A. flavus*, and *A. niger*, have been documented to cause respiratory diseases in humans [ 7 - 9 ]. *Aspergillus* spp. can release millions of spores daily, which can induce infections and chronic pulmonary disease in humans via inhalation [ 10 ]. These spores produced by fungi can survive for extended durations until environmental conditions become conducive for growth. It should be mentioned that dispersing these spores may lead to aspergillosis in susceptible individuals [ 11 ].

As a commonly utilized strategy, treatment using antifungal agents (such as itraconazole and ketoconazole) has some limitations due to systemic antibiotic consumption, the instability of oral drugs in aqueous intestinal fluids, and the main first-pass metabolism of CYP3A before entering the systemic circulation [ 12 ,  13 ]. All of these drawbacks are responsible for significant adverse effects and low bioavailability of the agents. Despite extensive investigations into the pathogenesis and prevention of pulmonary aspergillosis, as well as efforts to apply prophylactic interventions targeting this infection (e.g., regulation of the tracheal tube cuff and reduced sedation protocols), the burden of fungal infections remains a major concern in patients undergoing ventilation within intensive care units [ 14 ]. Given that the clinical presentation of pneumonia arises over several days following the establishment of infection, with incidence peaking around 7 days after the initiation of mechanical ventilation, a therapeutic window exists to curtail infectious colonization and precocious spread in the intervening period [ 15 ]. To overcome the problems associated with traditional treatment, pulmonary delivery of nano-formulations of antifungal drugs have become a focal point of research due to the advantages of targeted delivery.

Nanoparticles, with their tunable size and surface properties, can be engineered to enhance drug delivery directly to the site of infection within the alveolar space. This targeted approach facilitates increased drug concentrations at the fungal infection site, reduces systemic exposure, improves bioavailability, leads to slower clearance, and prolongs therapeutic effects. Furthermore, surface modification of nanoparticles can enable specific interactions with alveolar cells or the fungal pathogens themselves, enhancing drug uptake and antifungal activity. Consequently, nanoparticulate drug carriers represent a promising strategy for overcoming the challenges associated with treating pulmonary infections and improving patient outcomes.

### Nanocarriers for pulmonary delivery against fungal infection

Nanoparticles can serve as effective pharmaceutical delivery platforms for treating pulmonary mycoses due to their great surface area-to-volume proportion and capacity to augment the bioactivity of anti-mycotic drugs [ 16 - 18 ]. Given their typically small dimensions (below 100 nanometers), these formulations can permeate into alveolar cavities, thereby facilitating optimal anti-mycotic localization at the sites of pulmonary infection [ 19 ,  20 ]. Additionally, these nano-systems exhibit augmented permeation and retention, which can be useful for targeted delivery and controlled release via increased solubility, protection against the degradation of enzymes, enhanced blood circulation time, and increased absorption by epithelial cells [ 21 ,  22 ]. These properties enable lower doses of antifungal drugs to be administered, thereby reducing whole-body exposure to the pharmacological agents, which can potentially decrease the risk of adverse reactions linked to azole therapy, such as hepatotoxicity [ 23 ,  24 ]. Nanocarriers may consist of lipid or polymeric constituents, but lipid-based platforms are preferentially considered due to their greater physiological compatibility and biodegradability over time [ 25 ]. Nevertheless, a drug transportation system capable of surmounting pulmonary barriers is imperative to optimize the efficacy of pharmaceutical agents within respiratory tract epithelial cells.

In a promising study, lipid-polymer hybrid nanoparticles were coupled with nucleic acid cargo to tackle cystic fibrosis mucus secretions [ 26 ]. Results of the aforementioned study confirmed the ability of non-PEGylated lipid-polymer hybrid nanoparticles to overcome mucus barriers in the airway epithelium. Notably, PEGylation did not enhance mucus permeation in sticky and complex samples. Liposomal amphotericin B (AmBisome) was named the first U. S. Food and Drug Administration-approved polyene antifungal drug [ 27 ]. As a result of nanoliposome encapsulation, the pharmacokinetics of amphotericin B were altered, with prolonged circulation and selective transfer into fungal cells, thereby reducing nephrotoxicity. However, the conventional amphotericin B liposomal nano-formulation has a tendency for antifungal agent leakage from the carrier during blood circulation, major clearance of it by the reticuloendothelial system, and incapacity for extravasation into the infected epithelial cells.

In a recent study, it has been suggested that three preparation methods, including a solvent-free approach, a solvent evaporation procedure, and ethanol injection, be used to encapsulate amphotericin B in liposomes [ 28 ]. Valuable advantages of the solvent-free method include the removal of the evaporation stage, a major concern for industrial-scale manufacturers, and the production of a harmless final product in biosystems. The assessment based on transmission electron microscopy analysis indicated that after the lyophilization process, the liposomal amphotericin B preserved its spherical nanostructure without aggregation or deformations. In addition, storage in the refrigerator can enhance the stability of this powder. In contrast, previous articles have shown that liposomal amphotericin B is unstable over long periods in solution form, as it undergoes photolytic degradation, oxidation, and hydrolysis [ 29 ]. However, in the solvent-free procedure, after lyophilization, the size of the nanoparticles increased but remained within the size range considered appropriate for nano-liposomal delivery systems [ 29 ].

Table 1 presents a comparative analysis of four categories of nanomaterials for fungal infections.

**Table 1:** Comparison of characteristics and applications of various nanomaterials in the treatment of fungal infections.

Characteristics	Nano-graphene Oxide [ 31 - 33 ]	Liposomes [ 34 - 36 ]	Hybrid Nanoparticles [ 37 - 39 ]	Nano Metallic particles [ 40 - 42 ]
Composition	Graphene with oxygen-containing groups	Phospholipids and cholesterol	Lipid and polymer	Metals and metal oxides
Preparation	Oxidation of graphene	Encapsulation of drugs in spherical bilayers	Combination of lipid and polymer	Chemical or biological synthesis of metals
Production Cost	Moderate	Moderate	Variable	High
Shape characteristics	Nanoscale-thin sheets	Spherical lipid bilayers	Diverse shapes with functional groups	Various shapes depending on the type of metal
Particle Size	100-200 nm	50-300 nm	50-200 nm	1-100 nm
Stability	Unstable, requires lyophilization	Drug leakage possible	Good stability	Stability depends on the metal type
Mechanism of action in fungal infection treatment	ROS, pore formation in fungal walls (nano-knives), fungal cell encirclement blocking access to oxygen and nutrients, and biofilm inhibition. Synergistic effects when combined with certain antifungal drugs	Reduction of toxicity and enhancement of antifungal drug penetration	Enhancement of drug absorption and penetration to deep tissues	Oxidative stress, membrane disruption
Drug delivery capability	Drug delivery to the infection site, reducing side effects, and inducing targeted delivery	Improvement of drug delivery and release	Enhancement of drug transport to the injection site	Enhancement of drug delivery to the infection site
Characteristics	NGO	Liposomes	Hybrid Nanoparticles	Other Nanoparticles (Metallic)
Safety and biocompatibility	Toxicity at high concentrations, surface modification improves biocompatibility	Liposomal drug forms are less toxic than traditional forms	Usage of biocompatible materials can reduce toxicity	The variable depends on the metal and its preparation
Administration method	Inhalation	Mostly intravenous	Inhalation possible	Variable
Stability in solution	It may be unstable in solution, requires lyophilization	It may be unstable in solution, prone to leakage	Better stability, compared to simple nanoparticles	Variable
Penetration capability	Small size enables deep penetration into the lungs and infection sites	Limited penetration into deep tissues	Special coatings improve deep tissue penetration	Variable
Impact on the immune system	It may induce inflammation in some cases	Issues, like drug leakage and low stability	Compared to simple nanoparticles, better cell uptake, lung retention, and antifungal effects	Variable
Surface area	High	Moderate	Variable	Variable
Surface modifiability	High	Moderate	High	Moderate

NGO: nano-graphene oxide, ROS: reactive oxygen species

### Challenges associated with available anti-fungal liposomes

Up to now, several nano-liposomal antifungal formulations (such as amphotericin B) have been developed; however, these nano-formulations still suffer from problems, such as stability, lipid peroxidation, and cellular toxicity caused by nontargeted delivery of liposomes via the parenteral route [ 30 ]. In addition, the rapid removal of these nanoparticles from blood circulation caused by uptake by the reticuloendothelial system, with significant decomposition in the liver, is another factor that cannot be overlooked.

Notably, the treatment of fungal infections that are situated in the lower respiratory tract or penetrate further into the lung parenchyma can prove challenging [ 43 ]. This is due to the fact that maintaining therapeutically effective concentrations of an antifungal agent at the site of infection over an extended duration is often impossible. Rapid clearance of drugs from these highly vascularized pulmonary regions, where blood flow turnover is elevated, can limit the sustained presence of sufficient drug levels needed for optimal treatment efficacy. Locating infections in deeper recesses of the lung that are perfused by profuse circulation renders fungal therapeutic interventions more difficult.

In this regard, a nanoliposome-based inhaled system was designed to directly deliver antifungal drugs to the lungs, which can reduce drug levels throughout the blood while maintaining therapeutic concentrations at the focal of infection, thereby reducing nephrotoxicity, compared to intravenous administration. Inhalation therapy is employed for the local treatment of pulmonary diseases, including chronic obstructive pulmonary disease, pneumonia, bronchiectasis, asthma, cystic fibrosis, and aspergillosis.

Local inhalation of drugs targets the lungs for therapeutic effects, offering several advantages over other administration routes, including a large absorption area, rapid absorption due to a thin membrane, reduced enzyme activity that bypasses the first-pass effect of the liver, and the ability to maintain high drug concentrations at the target site. This method presents a non-invasive alternative to injections, allowing for lower doses and enhanced bioavailability [ 44 ].

Extensive research has indicated that common antimicrobial drugs, such as amphotericin B, amikacin, tobramycin, and rifampicin, which are often used to combat lower respiratory infections caused by pathogens, such as *Mycobacterium tuberculosis*, *Candida albicans*, and *Pseudomonas aeruginosa*, have serious side effects when administered intravenously or orally. However, when administered via inhaled drug delivery, their side effects are significantly ameliorated [ 45 ,  46 ].

A broad array of pulmonary fungal diseases, encompassing invasive aspergillosis, chronic pulmonary aspergillosis, *Aspergillus* bronchitis, and allergic bronchopulmonary aspergillosis, is predominantly attributed to *Aspergillus* spp., especially *A. fumigatus*. Regardless of their clinical manifestations, azoles serve as the first-line therapeutic approach [ 47 ].

Pulmonary inhalation is useful for drugs with limited oral absorption, such as amphotericin B, due to its low water solubility and low membrane permeability [ 45 ]. Additionally, due to the low metabolic activity in the cellular lung, pulmonary inhalation delivery is important for the delivery of medications that are sensitive to gastric conditions, such as pH and enzymatic activity, especially those prone to hepatic metabolism [ 45 ]. This parallels research demonstrating that liposomal dry powder inhalation aerosols designed for targeted lung delivery are advantageous for compounds vulnerable to gastrointestinal and liver enzymatic breakdown.

In theory, inhaled liposome formulations could provide sufficiently high concentrations at the site of infection that exceed the minimum inhibitory concentration (MIC) for multidrug-resistant pathogens and, therefore, reduce the likelihood of developing drug resistance [ 45 ]. As a result, inhaled liposome antibiotics may limit opportunities for pathogens to evolve strategies of resistance, since low concentrations below the MIC of a drug could lead to increased mutation rates and, hence, the emergence of resistant subpopulations of pathogens that cannot be eradicated. However, meticulous attention must be paid to formulation engineering to improve consistent bioavailability, as design considerations can significantly impact this parameter.

Various factors, such as the dissolution of antifungal aerosol nanoparticles in mucus and epithelial fluid, the appropriate diffusion of the formulation toward microorganisms at the mucosal site, the ability of the antibiotic against resistant mucoidal strains, and diffusion into spaces containing sputum, affect the clinical effectiveness of inhaled liposome antibiotics, especially in patients with cystic fibrosis. Delivery of liposomes via nebulization can lead to alterations in their physicochemical characteristics, and the deposition of the formulation within pulmonary alveolar macrophages may influence their functions [ 48 ]. In addition, the pulmonary branching structure impedes access to deeper lung regions, while clearance mechanisms efficiently remove inhaled drugs [ 45 ]. Given that most chronic lung infections occur in the airways of the lung lumen, effective delivery of pulmonary drugs requires advanced aerosol formulation methodologies and multifaceted delivery systems. Therefore, administering treatments directly to the lungs is a challenge due to the intricate anatomical and physiological architecture of the respiratory tract.

### Comparison of nanoliposome-based inhaled systems and inhaled hybrid nanoparticles

Many studies compared the performance of hybrid nanoparticles and that of plain nanoparticles, which have been used in pulmonary antibiotic delivery [ 49 ]. Results of various investigations have demonstrated that hybrid nanoparticles have greater cellular uptake, improved lung retention, and enhanced antifungal efficacy, compared to plain nanoparticles as a platform for pulmonary drug delivery [ 50 ,  51 ].

In a study, nebulized hybrid nanoparticles (dipalmitoyl phosphatidylcholine-coated chitosan nanoparticles) of voriconazole were evaluated for pulmonary aspergillosis, and the results were compared with those of the plain drug and nanoparticles [ 52 ]. Coating nanoparticles with the lung-specific phospholipid dipalmitoyl phosphatidylcholine exhibited augmented biocompatibility and suitable interaction with lung surfactant monolayers since this phospholipid constitutes approximately 50-60% of pulmonary surfactant [ 53 ].

Furthermore, the presence of this phospholipid minimizes macrophage cell uptake, thereby facilitating nano-formulation diffusion and internalization into the alveolar epithelial cells. In addition, modification of chitosan nanoparticles with lung surfactant lipid, dipalmitoyl phosphatidylcholine, can be postulated to hinder the adhesion of nano-formulation with the mucus barrier and escape deposition in the upper respiratory system [ 54 ]. This is followed by nanoparticle integration with pulmonary surfactant monolayers, which leads to interaction with alveolar epithelial cells and improves their internalization via cellular pathways, thereby enhancing the residence time of the drug in the lungs. Findings of the present study confirmed previous reports demonstrating heterodispersity arising from the generated aerosol cloud [ 55 ,  56 ]. However, it was noteworthy that the nanoparticles exhibited good size and structural integrity preservation within the nebulized droplets, without any discernible aggregation. Although the researchers claimed that this hybrid nanoparticle accumulated in the cytoplasm of pulmonary epithelial cells, evaluation of cellular uptake analyses revealed that internalization of lipid-polymer hybrid nanoparticles in Calu-3 and A549 cell lines increased with dose and time.

### Nano graphene-based materials

After the discovery of graphene, nano-graphene oxides (NGOs) received unique attention to achieve better attributes in comparison to their counterparts [ 57 ]. The functional groups (hydroxyl, carbonyl, and epoxy) on the surface of NGO provide the potential for utilization in different biological applications, including antifungal, antibacterial, bioimaging, tissue engineering, drug delivery, and antimicrobial applications [ 58 ,  59 ]. The outstanding solubility of NGOs in different solvents can be attributed to the presence of these functional groups on the surface of GO nanosheets [ 60 ].

NGO can be functionalized with a diverse range of biomolecules, including antibodies, genes, DNA, and RNA, to enhance their targeted delivery to the intended site of action [ 61 ]. Notably, it was observed that they exhibited potent antimicrobial activity through the mechanism of oxidative stress [ 62 ]. Table 2 summarizes some studies related to the effects of nanographene on fungi. Graphene and its derivatives are antimicrobial agents that can be utilized in the treatment of antibiotic-resistant infectious diseases, and they are considered safe for use in humans. NGOs can show a variety of antimicrobial activities, including antifungal properties, as discussed in the subsequent sections. Table 3 shows the effect of nano-graphene oxide in the treatment of fungal infections.

**Table 2:** Summary of studies related to the effects of nanographene on fungi.

References	Type of nanoparticle	Type of fungi	Fungal infections or target organs	Main results
Wagner et al. 2017 [ 63 ]	Polyethylene glycol-functionalized poly (lactic acid-co-glycolic acid) and graphene oxide nanoparticles	*Candida albicans*	Vaginal Candidiasis	Nanoparticle drug-delivery vehicles may exacerbate inflammation in active yeast vaginal infections by increasing inflammatory recruitment.
Diez-Orejas et al. 2018 [ 64 ]	GO	*C. albicans*	Systemic Candidiasis (bloodstream)	GO uptake by macrophages modulates their phagocytic capability and increases the clearance of dead microorganisms during infection.
Nguyen et al. 2019 [ 7 ]	G and GO	*Aspergillus niger* and *Aspergillus flavus*	Aspergillosis (Lungs, Sinuses)	Examined the toxic effects of graphene and graphene oxide on *A. niger* and *A. flavus*, revealing a 62% reduction in biomass and altered hyphae, with *A. niger* being more affected by GO and *A. flavus* by G.
Kahsay et al. 2020 [ 65 ]	GO	*Trichophyton mentagrophytes* and *C. albicans*	Dermatophytosis (Skin, Nails) and Candidiasis (Oral, Vaginal)	rGO/Fe3O4 NCs showed antifungal activities against *T. mentagrophytes* and *C. albicans*
Wang et al. 2021 [ 66 ]	GO	*Fusarium graminearum*	*Fusarium* Head Blight (Cereal Crops, Agricultural)	The strong synergistic activity of GO with existing fungicides demonstrated the great application potential of GO in pest management.
Zhang et al. 2022 [ 67 ]	GO	*Bipolaris sorokiniana*	Leaf spot disease (plants)	Inhibitory effect of GO on *B. sorokiniana* was primarily related to the destruction of the cell membrane.
Gottardo et al. 2023 [ 68 ]	Reduced graphene oxide (rGO)	*Candida* species, Epidermophyton, *Microsporum*, and *Trichophyton* species (Dermatophyte agents)	Candidiasis (nails, skin) and Dermatophytosis (skin, nails, scalp)	Both yeast and dermatophytes clinical isolates were inhibited at a minimum of 6 and 24 h, respectively, but after 2 and 12 h, they had initial antifungal interference, respectively
Selvi et al. 2024 [ 69 ]	Silver-decorated graphene oxide (Ag/GO)	*Candida albicans*, *Candida krusei*, and *Candida tropicalis*	Systemic and cutaneous candidiasis	*In-vitro* antifungal activity of Ag/rGO nanocomposite revealed a higher zone of inhibition against *C. albicans*.

Graphene oxide (GO), Reduced graphene oxide (rGO)

**Table 3:** Applications of nano-graphene oxide in treating fungal infections.

Advantages	Mechanism of Action	Application
Rapid action and reduced drug usage	Forms a protective layer on the skin and inhibits fungal growth.	Treatment of superficial fungal infections
Reduced side effects	Delivers drugs directly to infection sites and enhances drug efficacy.	Treatment of systemic fungal infections
Early detection of infections	Acts as a contrast agent in imaging techniques.	Diagnosis of fungal infections

G: graphene, GO: graphene oxide

### Factors affecting the antifungal activity of nano-graphene oxide

In most NGO studies, notable distinctions have been observed in the antifungal activities between NGO nanosheets and graphite [ 70 ,  71 ]. The significant antifungal effects of this material depend heavily on the concentration and duration of exposure [ 72 ]. Furthermore, as the percentage of functional groups increases and the size decreases, the toxicity effect increases, leading to a higher impact on fungal killing [ 73 ,  74 ]. Research findings have indicated that while conductive reduced graphene oxide exhibits a significantly higher oxidation capacity, it is NGO layers with smaller lateral size dimensions that demonstrate superior antifungal properties. Moreover, the thickness of NGO layers and the angle of orientation with microorganisms may affect the anti-microbial efficiency [ 75 ]. Table 4 tabulates the characteristics of the NGO used in the treatment of fungal infections.

**Table 4:** Advantages and disadvantages of using nano-graphene oxide in treating fungal infections.

Disadvantages	Advantages
Potential toxicity: At high concentrations, NGO can be toxic to human cells [ 76 ]	Strong antifungal effects: oxidative stress, membrane damage, fungal encapsulation, inhibition of fungal proliferation, and biofilm disruption [ 62 ]
Structural changes: An NGO may exhibit structural changes when exposed to environmental conditions, such as photodegradation, oxidation, and hydrolysis, which can affect its performance [ 77 ]	Drug delivery capability: enhances drug efficacy, reduces side effects, and allows for lower drug doses [ 78 ]
Accumulation and inflammation: Administration methods, like intratracheal injection or inhalation, may lead to NGO accumulation and increased inflammation in the lungs [ 77 ]	Therapeutic targeting enables specific penetration to the infection site. Inhalation can directly deliver the drug to the lungs and minimize systemic side effects [ 79 ]
Instability: The NGO may be unstable in solution and require lyophilization to maintain its structure [ 77 ]	Overcoming drug resistance: It increases drug concentration at the infection site, reducing opportunities for pathogen mutation and aiding in overcoming drug resistance [ 80 ].
Body clearance: The NGO may be rapidly cleared from the bloodstream by the reticuloendothelial system and accumulate in the liver [ 81 ]	Enhanced biocompatibility: Surface modifications (e.g., polymer coatings) can increase the biocompatibility of the NGO [ 82 ]
Uncertainty in mechanisms: The Exact mechanisms of NGO action in the respiratory system are not fully understood [ 79 ]	High modifiability: NGOs, with various functional groups, can be modified and conjugated with biomolecules, such as antibodies, genes, and antifungal drugs [ 83 ]
Industrial production challenges: Industrial-scale production of NGO with consistent and desirable properties still faces challenges [ 84 ]	Synergistic effect: The Combination of NGO with antifungal drugs can create synergistic effects, enhancing therapeutic efficacy. Reduced drug dosage: It allows for lower drug doses, reducing side effects [ 83 ]

### Antifungal mechanisms of nano-graphene oxide

**Figure 1:**
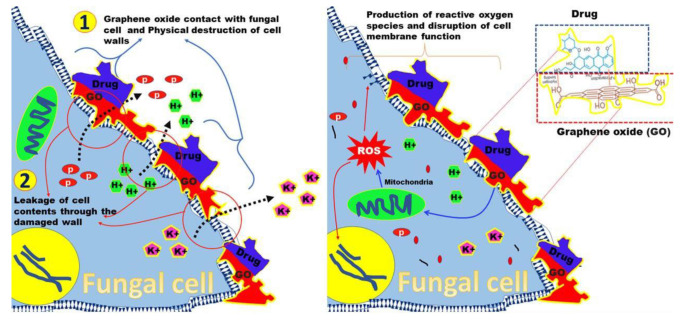
Functions and mechanisms of graphene oxide nanoparticles against fungi.

It has been reported that the NGO demonstrates favorable inhibitory effects against fungi (Figure 1). This activity is likely attributed to the ability of NGOs to disrupt microorganism membranes through direct interactions with inorganic biomolecules. It is challenging to understand the interaction between GO and fungi due to the divergent characteristics of GO, such as morphology, oxidation degree, size, hydrophilicity, number of sheets, and the presence of nanocomposites. Multiple mechanisms are reported in the literature, including oxidative stress, nano knives, plasma membrane damage, wrapping, apoptosis, necrosis, and inflammatory response [ 76 ,  85 ,  86 ]. In the nano-knives mechanism, the NGO causes degradation of membrane integrity in microorganisms due to pores on the NGO surface and the sharp edge properties of graphene-based materials [ 62 ,  87 ,  88 ]. Another significant mechanism of NGO with fungi is oxidative stress. Oxidative stress arises from an imbalance between the generation and removal of reactive oxygen species (ROS), resulting in cells being unable to effectively manage the accumulated oxidative damage through their intrinsic repair mechanisms. Remarkably, certain metal oxides within the composite of NGO contribute to the enhancement of ROS formation in the presence of light [ 89 ,  90 ]. The occurrence of oxidative stress leads to the degeneration of the cell membrane and initiates the processes of apoptosis and necrosis [ 91 ]. Regarding the interactions between graphene-based nanomaterials and fungi, the majority of research endeavors have concentrated on enhancing the anti-fungal properties of NGOs through their modification with peptides, anti-mycotic drugs, and metals [ 92 - 94 ]. The GO nanocomposites, which are composed of polymers, including chitosan, exhibit antimicrobial properties by promoting the production of ROS [ 62 ,  95 ]. The antifungal mechanism in NGO-Ag composites involves the destruction of respiratory cells by Ag+ ions through the contact-killing mode [ 68 ,  96 ]. Notably, a monolayer of NGO, which does not have any pores, possesses a structure that restricts the passage of nutrients, such as oxygen and carbon dioxide [ 97 ]. As a result, even if a molecule is permeable, microbial proliferation is effectively inhibited due to the encapsulation of the microorganism within a highly impermeable environment [ 86 ,  98 ]. In this process, the exposure time plays a crucial role in determining the antifungal efficiency. However, there is limited understanding regarding the precise fungal response to the presence of NGO in the respiratory system, including physiological alterations and the activation of toxicity pathways.

### Cyto and genotoxicity of nano-graphene oxide

Suitability of pulmonary-targeted delivery using graphene oxide is determined by their increased efficacy, stability, and, most importantly, its toxicity and safety profile. Nano-graphene oxide can be considered an acceptable example of a safe design. Despite the potential benefits, the toxicity associated with inhalation exposure to this nanomaterial has not been fully elucidated. Towards this goal, Han et al. reported that the acute inhalation toxicity associated with NGO was low, while the *In Vivo* animal model studies demonstrated potential reproductive genotoxicities [ 99 ,  100 ].

In a recent study, the NGO was administered via inhalation to male rats to examine their short-term *In Vivo* biodistribution [ 72 ]. According to their results, no remarkable systemic toxic effects were observed in the bronchoalveolar lavage cells, including the lymphocytes and macrophages. The results also exhibited a similar trend to those of a previous NGO exposure study [ 101 ]. Contrary to these results, another study using nano-graphene with sizes of <2, 5, and 20 µm administered via pharyngeal aspiration, reported increased inflammatory markers in the bronchoalveolar lavage fluids [ 102 ]. Such differing outcomes may be attributed to the varying size distributions of graphite nanoplates used across the studies. The NGO with a lateral size of 200-500 nm has been shown to induce cyto-genotoxic effects in human lung fibroblasts in a dose-dependent manner (1-100 μg/ml) via the mechanism of oxidative stress and the surface charge properties of NGO [ 103 ]. On the cellular level, an NGO with a lateral size range of 160-780 nm did not penetrate A549 cells but did induce oxidative stress in a dose-dependent manner and resulted in a slight decrease in cell viability at high concentrations, suggesting that NGO may be a safe material [ 104 ]. Additionally, the choice of delivery methods, such as pharyngeal aspiration techniques or inhalation of aerosolized materials, can also influence the inflammatory response. When pharyngeal aspiration and/or intratracheal instillation techniques are used to administer nano-carriers for drug delivery to the lungs, it should be considered that they can lead to increased aggregation of the nanostructures.

In a preclinical study, the intratracheal instillation of NGO in male Sprague-Dawley rats using a nose-only inhalation exposure system showed no significant inflammatory response [ 72 ]. In contrast, another study reported an increased inflammatory response, including an elevated acute phase response and elevated serum amyloid A levels, in mice following the intratracheal instillation of NGO and reduced graphene [ 105 ].

In a study, the genotoxicity of reduced graphene oxide nanoribbons in human mesenchymal stem cell lines was measured to assess the extent of DNA damage. According to their results, nanoparticles demonstrated genotoxicity at a 1.0 μg/mL concentration with DNA fragmentations and chromosomal aberrations [ 106 ]. Moreover, pristine NGO cytotoxicity toward bone marrow-derived mesenchymal stem cells was reported to be concentration-dependent [ 107 ]. These findings confirm the important role that the shape and dispersion status of graphene nanomaterials play in their interaction with cell lines and the subsequent induction of toxicity.

### Biocompatibility of nano-graphene oxide

The NGO can be loaded into a kind of biodegradable polymeric composite to improve its physicomechanical properties [ 108 ]. Although these polymer-hybrid graphene materials are utilized in antimicrobial applications, they are not expected to compromise biocompatibility; rather, they are expected to facilitate the reduction and suppression of microbial growth [ 109 ]. Several studies have reported a decrease in fungal viability when exposed to NGO in nanocomposites [ 110 ]. Various physicochemical characteristics of NGO, including ROS, layer number and thickness, and sharp edges, all contribute to this decline.

Furthermore, the concentration of NGO and the exposure time can enhance the antifungal activity and negatively affect biodegradability and biocompatibility.

Various *In Vivo* and *in vitro* studies have been used to understand the biocompatibility of NGO-based compounds. In one study, poly (acrylic acid) and poly (ethylene glycol) were used to cover the NGO at different doses. The results obtained in their study indicated the extraordinary biocompatibility of poly (acrylic acid), compared to PEGylation for the functionalization of NGO [ 111 ].

In another study, NGO-modified chitosan was synthesized, and its antifungal activity against *Fusarium graminearum* was investigated [ 112 ]. *In vitro* studies reported that the combination of NGO and chitosan exhibited a notable synergistic inhibitory effect on the mycelial growth of *F. graminearum*, compared to a single NGO or chitosan. According to *In Vivo* studies, the NGO-chitosan composite demonstrated a significant reduction in both the incidence and severity of the disease, compared to a single NGO or chitosan, and the control efficacy of the NGO-chitosan composite reached 60.01%. In an unprecedented study, for the first time, the fungicidal activity of hybrid nano-compounds based on silver/reduced GO )rGO/Ag( was tested against clinical isolates of *Candida* spp. [ 68 ]. The antifungal susceptibility assay was conducted using the microdilution procedure based on modified guidelines from the Clinical and Laboratory Standards Institute. The highest inhibitory activity against *Candida* species was observed in rGO/Ag12% at a minimum of 6 h. However, after 2 h, initial antifungal interference was observed. None of the hybrid formulations of rGO/Ag nanocomposites exhibited toxicity towards *G. mellonella*. The conclusions are of paramount importance for clinical studies. *Candida albicans* is the predominant pathogenic fungus frequently isolated in nosocomial infections among immunocompromised patients [ 113 ]. The ability of this fungus to convert from yeast to hyphal morphology plays an important role in the formation of resistant biofilms, which effectively protect *C. albicans* against the effects of antifungal agents [ 114 ]. Recently, a study was conducted to investigate the effectiveness of zinc oxide nanorods decorated on an NGO against the human pathogen *C. albicans* [ 93 ].

The results obtained in their study demonstrated a significant reduction in the viability of *C. albicans* yeasts and an impact on hyphal development. The toxicity of NGO-Zinc oxide nanorods in HaCaT human keratinocyte cells showed that the nanomaterial was not toxic when glycerol was added as an emulsifier to the suspensions. The outcomes illustrated that NGO-Zinc oxide induced mortality in yeast cells via ROS formation, indicating its potential as an antifungal nanomaterial with extraordinary biocompatibility on human cells.

In another study, the synergistic antifungal potential of NGO-ethylene diamine tetraacetic acid (EDTA) and NGO-chitosan was evaluated against *C. albicans* [ 74 ]. The *in vitro* zone of inhibition outcomes verified that the nanocomposites showed inhibitory activity against *C. albicans* (16.0 mm for NGO-chitosan and 22.0 mm for NGO-EDTA). Notably, fungal cells may ultimately be damaged when they interact with NGO composites due to several mechanisms, including oxidative stress, wrapping/isolation, and membrane stress.

A novel approach for antifungal activities is the reaction of biocompatible NGO composite with amino acids, including Methionine and Cysteine in the presence of cellulose [ 115 ]. The method used for the formation of these antimicrobial nanocomposites involved two steps: the first step was the periodate oxidation of cellulose, and the second step was the reaction with amino acids in the presence of NGO. Both dialdehyde cellulose/NGO/Met and dialdehyde cellulose/NGO/Cys exhibited promising antifungal activity against the unicellular fungi *C. albicans* and *Cryptococcus neoformans*.

In an exciting study, a ternary NGO composite was prepared using chitosan and cerium oxide as a therapeutic platform against fungal and bacterial infections as well as breast cancer [ 116 ]. The results indicated the effective therapeutic behavior of the NGO/chitosan/cerium oxide composite against *Aspergillus niger*, *Escherichia coli*, *Staphylococcus aureus*, and *Salmonella* species. Furthermore, the NGO/chitosan/cerium composite exhibited outstanding anti-cancer potential on the MCF-7 cell line, with only 18.8% of cells remaining viable when exposed to the maximum concentration of 1,000 µg/mL of the nanocomposite. In addition, cerium oxide incorporation with chitosan enhanced their interaction and their reactivity.

### Antifungal drugs delivery by nano-graphene oxide

The NGO is a material with amphiphilic properties, possessing a unique high surface area, which makes it a promising functional excipient for efficient drug loading and targeting. Recently, a study was conducted to investigate the antifungal activities and clotrimazole drug release profiles of NGOs incorporated into a buccal polyelectrolyte film [ 117 ]. The mucoadhesive excipients alginate and chitosan were chosen to create a polyelectrolyte film incorporating clotrimazole. According to their findings, the inclusion of NGO in the formulations resulted in a significant release of clotrimazole from the buccal films at pH 6.8, which could potentially be attributed to NGO modifying the electrostatic interactions between alginate and chitosan. The researchers have claimed that the antifungal activity against *C. albicans* was significantly higher in the formulation containing 0.04 wt% NGO, compared to the formulation without NGO. Nonetheless, when the NGO concentration increased to 0.09 wt%, the antifungal efficacy of the films was similar to that of the films with 0.04 wt% NGO, implying that the hydrophobic and electrostatic interactions between clotrimazole and NGO may also modulate the antifungal activity of clotrimazole. A similar study employed 1 wt% NGO in the formulations to evaluate the effect of NGO on the release and antifungal activity of clotrimazole from chitosan/alginate-based foams *in vitro* and affirmed the lower amount of NGO for enhancing the antifungal effect [ 95 ].

Recently, a novel formulation of NGO/fluconazole has been developed to examine its antifungal activity against fluconazole-resistant *C. albicans* [ 94 ]. The results indicated that the NGO/fluconazole formulation with minimal cytotoxic effect on the SW480 cell line was suitable for drug release in the Flui phosphate-buffered saline medium (72.42%). Notably, this formulation could enhance the antifungal effect against fluconazole-resistant *C. albicans* through DNA fragmentation, compared to NGO and fluconazole. Other studies have shown that incorporating fluconazole with different drug delivery systems, such as PEG-gold nanorods and cholesterol-gold nanorods, enhances the anti-mycotic effect of the drug against various strains of *C. albicans* [ 118 ]. However, the loading efficiency percentage of fluconazole on the NGO was higher (72.42%), compared to PEG-gold nanorods (62.5%) and cholesterol-gold nanorods (33.0%). Cholesterol/fluconazole/gold nanorods exhibited higher antifungal activity with a MIC of 0.25 nM, compared to that of PEG/fluconazole/gold nanorods (with MIC of 0.125 nM). The observed difference in antifungal activity can be attributed to the pattern of fluconazole release from formulations.

In a study, *in vitro* and *In Vivo* experiments were carried out to investigate whether NGO-composite containing indolicidin inhibited *C. albicans* yeasts [ 92 ]. This formulation exhibited favorable antifungal properties (MIC=3.12 μg/mL), compared to indolicidin (MIC 12.5 μg/mL) and fluconazole (MIC 4 μg/mL) in the cell line model. In *In Vivo* experiments, the NGO-indolicidin formulation eliminated the *C. albicans* infection in the liver and spleen of BALB/c mice, similar to fluconazole (*p*=0.001). Notably, incorporating indolicidin with gold nanoparticles appeared to reduce the antifungal activity of indolicidin, compared to the NGO-containing indolicidin formulation against fluconazole-resistant strains of *C. albicans* (MIC≥4) [ 119 ]. In contrast, no significant inhibitory effect of this formulation was observed, compared to fluconazole and amphotericin B on standard strains of *Aspergillus* spp. [ 120 ]. These outcomes are consistent with the fact that the integrity of the *Aspergillus* cell wall can prevent nanocomposite penetration. In a preliminary study, an *in vitro* experiment was carried out to investigate whether Amphotericin-B-loaded polymer-NGO was effective against *Leishmania amazonensis* cells after chemo-photothermal therapy [ 121 ].

Antiproliferative effects of the NGO-polyethyleneimine-Amphotericin-B formulation exhibited superior efficacy against the parasitic cells, compared to NGO-polyethyleneimine, which this activity was further potentiated under near-infrared (NIR) light irradiation. The scanning-transmission electron microscopy analysis of *L. amazonensis* promastigotes cells provided evidence indicative of the induction of autophagic and necrotic pathways leading to cellular demise. In contrast, no significant differences were observed in drug release from the NGO-Pluronic® P123- P123-Amphotericin-B formulation in the presence and absence of NIR light. In addition, cytotoxicity studies showed that the NGO-polyethyleneimine-Amphotericin-B formulation was nontoxic to mammalian host macrophages, whereas rGO-Pluronic® P123-Amphotericin-B was very toxic. Therefore, the facile synthesis, excellent photothermal effect, and extraordinary loading capacity make the NGO platform a promising Candidate for topical therapy.

### Remarks and conclusions

To date, considerable research has been conducted to develop targeted therapies for the effective treatment of fungal diseases. Fungal infections present significant risks to human health, particularly for immunocompromised patients [ 122 ,  123 ]. Furthermore, the increasing resistance of fungi to conventional antifungal agents heightens the urgent need for innovative treatment approaches. Understanding the biology of fungi is very important in order to identify therapeutic targets, which include exploring unique cellular pathways and structures that are present in fungi but not in human cells [ 124 ,  125 ]. The main challenge is to develop drug delivery systems with maximum efficacy on fungal cells while minimizing toxicity on human cells; therefore, the use of medical nanotechnology is a suitable option. By using its products, it is possible to increase the effectiveness of the treatment while concurrently reducing side effects through the direct and targeted delivery of antifungal agents to the site of infection [ 126 ,  127 ].

Insufficient delivery of the drug to the site of infection, where colonies of fungal cells remain intact, may inevitably lead to incomplete eradication of all unhealthy cells and result in the failure of the treatment modality, as well as further invasion and relapse of the disease [ 128 ]. The homogeneous diffusion of drug molecules within the site of infection is an essential step that is made possible by overcoming the integrity of the fungal cell wall [ 129 ]. Given that the major purpose of a fungal therapy strategy is to enhance the effectiveness of treatment modalities, a synergistic combination of drug delivery can be considered a vigorous approach that may guarantee the success of therapy [ 130 ].

Therefore, the appropriate response of these nanosystems to specific fungal stimuli, such as pH changes or special enzymes, requires proper engineering [ 131 ]. This engineering involves a multidisciplinary approach, including molecular biology, medical mycology, and pharmacology, to improve the ability to transport and protect controlled antifungal agents, increase treatment efficacy, overcome drug resistance, and ultimately minimize the risk of toxicity associated with high doses of drugs to achieve successful outcomes [ 132 ].

Although one of the main problems associated with currently used antifungal treatments is the inevitable emergence of adverse reactions, targeted drug delivery may potentially resolve the issue of side effects to some extent [ 133 ]. The NGOs appear to be promising nanocarriers for antimicrobial drugs due to their specific characteristics that contribute to the synergistic effect [ 126 ].

## Conclusion

In conclusion, the utilization of an NGO as an innovative drug carrier presents a promising outlook for the treatment of pulmonary fungal infections. The unique physicochemical properties of this material, such as the generation of ROS, disruption of fungal cell membranes, and the capability to load antifungal drugs at lower doses, can enhance therapeutic efficacy while reducing side effects. However, challenges, such as potential toxicity, the need for surface modifications to improve biocompatibility, and an incomplete understanding of its mechanisms of action in the respiratory system warrant further investigation.

Overall, NGO, due to its potent antifungal properties, ability to deliver targeted drugs, and potential to mitigate drug resistance, is considered a promising Candidate for treating drug-resistant fungal infections. Nevertheless, comprehensive research on the design of drug delivery systems and the evaluation of biocompatibility under clinical conditions is essential to ensure the safe and effective application of this technology. 
